# Description of *Hemienchytraeus wuhanensis* sp. nov. (Annelida, Clitellata, Enchytraeidae) from central China, with comments on species records of *Hemienchytraeus* from China

**DOI:** 10.3897/zookeys.1015.59019

**Published:** 2021-02-04

**Authors:** Juanjuan Chen, Rüdiger M. Schmelz, Zhicai Xie

**Affiliations:** 1 The Key Laboratory of Aquatic Biodiversity and Conservation, Institute of Hydrobiology, Chinese Academy of Sciences, Wuhan, 430072, China Institute of Hydrobiology, Chinese Academy of Sciences Wuhan China; 2 IFAB, Institute for Applied Soil Biology, Hamburg, Germany University of Chinese Academy of Sciences Beijing China; 3 University of Chinese Academy of Sciences, Beijing, 100039, China Institute for Applied Soil Biology Hamburg Germany

**Keywords:** DNA barcoding, Oligochaeta, new species, taxonomy

## Abstract

*Hemienchytraeus wuhanensis***sp. nov.** is described from hardwood forest soil in Wuhan, China. This moderately sized enchytraeid species of 6–9 mm body length is characterized by: (1) an oesophageal appendage with tertiary branches, (2) three pairs of secondary pharyngeal gland lobes in V, VI, VII, (3) five pairs preclitellar nephridia, from 5/6 to 9/10, (4) dorsal vessel originating in clitellar segments, (5) a girdle-shaped clitellum, (6) a relatively small male reproductive apparatus without seminal vesicle, and (7) spermathecae that extend to VI–VII. DNA barcodes of paratype specimens of the new species are provided. Previous species records of *Hemienchytraeus* from China are critically discussed.

## Introduction

*Hemienchytraeus* Černosvitov, 1934 is a well-defined genus mainly distributed in the tropical and subtropical regions (Healy 1996; Xie et al. 1999; Schmelz and Römbke 2005). In Enchytraeidae it belongs, according to a molecular phylogenetic analysis (Erséus et al. 2010), to a clade separate from most other genera, but together with *Achaeta* . The genus is distinguished by the following characters: (1) head pore on prostomium; (2) two chaetae per bundle; (3) oesophageal appendage unpaired in III dorsally, behind pharyngeal pad, bifurcating into two primary branches, each of them usually branching into two or more secondary branches, and these sometimes with tertiary branches; (4) nephridial anteseptale large, with coils of canal; (5) no intestinal diverticula; (6) spermathecae free, blind-ending, ampulla without diverticula; (7) sperm funnel usually tapering distad (Schmelz and Römbke 2005; Schmelz and Collado 2010).

To date, 24 species have been reported worldwide (Schmelz and Römbke 2005; Dózsa-Farkas and Hong 2010; Schmelz et al. 2015). These species are mainly distributed in America (12 species), Asia (11 species), Africa (3 species), and Europe (2 species). Six species have been reported from China so far: *H. stephensoni* Cognetti, 1927, *H. bifurcatus* Nielsen & Christensen, 1959, *H. loksai* Dózsa-Farkas, 1989, *H. theae* Prabhoo, 1960, *H. planisetosus*Xie et al., 1999, and *H. brachythecus*Xie et al., 1999 (Wang and Cui 2007). Of these, the latter two are only known from China. In this paper, we add a new member to this list, which was collected from Wuhan, China. We describe the morphology of the species and compare it with congeners. We also provide COI sequences of *Hemienchytraeus wuhanensis* sp. nov. and calculate genetic distances using the sequences of *Hemienchytraeus* spp. available in GenBank. Finally, we comment on species finds of *Hemienchytraeus* spp. in China.

## Materials and methods

Soil samples were collected at forest sites at the Huazhong Agricultural University and Wuhan University, Wuhan, in April 2019. The samples were directly scooped using a steel shovel to a depth of ca 15 cm, placed in a breathable cloth bag and taken to the laboratory and stored at 4 °C. Worms were extracted from soil using a standard hot wet funnel extracting device (O’Connor 1962; Healy and Rota 1992). All worms were examined and identified alive. Body size, colour, movement, and maturity were observed with a Zeiss Stemi 508 stereomicroscope. Other characters were examined, measured, and photographed with a Zeiss Axio Imager A2 microscope using differential interference contrast optics and a Zeiss Axiocam 305 color digital camera with ZEN 2011 Blue Version software. The specimens were then anaesthetized in 30% ethanol and preserved in 75% ethanol (Dózsa-Farkas and Hong 2010). For taxonomic observation, some mature specimens were stained with borax-carmine, dehydrated in an ethanol series from 70% to absolute, mounted temporarily in clove oil and permanently mounted in neutral balsam (Dózsa-Farkas et al. 2015; Zhang et al. 2018). Drawings from whole mounts were made with the help of an Olympus drawing tube. Type material is deposited in the Museum of Aquatic Organisms (MAO), Institute of Hydrobiology, Chinese Academy of Sciences, Wuhan, China.

Total genomic DNA was extracted from five entire individuals respectively, using TIANamp Micro DNA Kit (Tiangen Biotech, Beijing, China). The COI gene was amplified from each DNA extract with primers LCO1490 (5'-GGTCAACAAATCATAAAGATATTGG-3') and HCO2198 (5'-TAAACTTCAGGGTGACCAAAAAATCA-3') (Folmer et al. 1994). These five specimens, of which no morphological parts are left, are part of the type series, as paratypes. Eight COI gene sequences of four different species in genus *Hemienchytraeus* were downloaded from GenBank, alignments were trimmed (resulting alignments were 591bp), aligned and K2P genetic distances were calculated using MEGA-X (Kumar et al. 2018).

Unless specified otherwise, measurements refer to mature fixed specimens (both whole-mounts and dissected specimens). When “*in vivo*” is given, measurements refer to living specimens.

## Taxonomy

### Hemienchytraeus wuhanensis sp. nov.

Taxon classificationAnimaliaEnchytraeidaEnchytraeidae

DF3E280F-F936-51FB-8418-CCB6BA1ACB36

http://zoobank.org/D3137BCA-E1CC-4FC7-AA55-A88FA9ED06E6

#### Holotype.

Fully mature, whole-mounted specimen, stained, HBO201904002.

#### Type locality.

**Figure 1.**
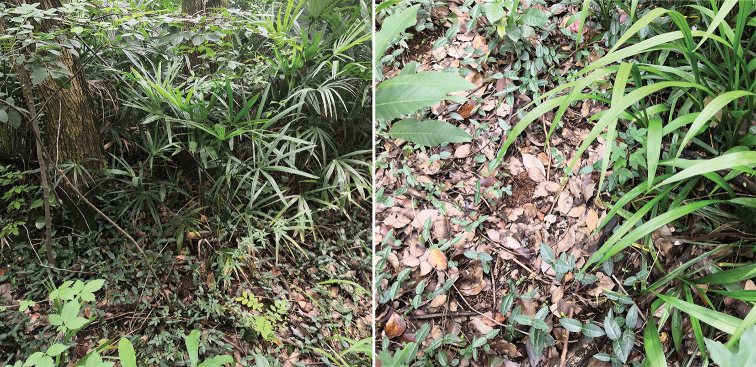
Habitat of *Hemienchytraeus wuhanensis* sp. nov., Mount Shizi, Huazhong Agriculture University, Wuhan, Hubei Province, China.

Mount Shizi, litter layer of hardwood forest (30°28'42.57"N, 114°21'10.48"E; 44 m a.s.l.), Huazhong Agricultural University (Fig. 1), Wuhan, Hubei Province, 6 April 2019, coll. Y. H. Ge.

#### Paratypes.

HBO201904003, HBO201904004 two whole-mounted fully mature specimens, HBO201904005–HBO201904007, three adult specimens, used entirely for DNA extraction; HBO201904008–HBO201904010 three adult specimens from the type locality maintained in 75% alcohol, same data as holotype. HBO201904001 one whole mounted fully mature specimen, HBO201904011–HBO201904012 two adult specimens used for extract DNA, and HBO201904013–HBO210904015 three adult specimens maintained in 75% alcohol from Mount Luojia, under a pine tree (30°32'05.39"N, 114°22'10.95"E; 31 m a.s.l.), Wuhan University, Wuhan, Hubei Province, 2 April 2019, coll. X. K. Jiang & J. J. Chen.

#### Etymology.

Named after the city where the species was found.

#### Distribution and habitat.

Mineral soil and organic layers under camphor trees near a narrow, tarred road at Mount Shizi, Huazhong Agricultural University; mineral soil and organic layers under pine trees at Mount Luojia, Wuhan University. The two hills are about 10 km apart, with little human disturbance.

#### Diagnosis.

This new species can be recognized by the following combination of diagnostic traits: (1) chaetae anteriorly and posteriorly of about the same size, not enlarged in caudal segments; (2) oesophageal appendage with tertiary branches; (3) three pairs of secondary pharyngeal gland ventral lobes in V, VI, VII, small in VII; (4) five pairs of preclitellar nephridia in 5/6–9/10; (5) dorsal vessel originating in clitellum segments; (6) clitellum girdle-shaped; (7) seminal vesicle absent; (8) spermathecae extending to VI–VII, not enlarged.

#### Description.

***Length*** 6.5–9.3 mm (*in vivo*), ***diameter*** 0.3–0.4 mm (*in vivo*) at clitellum. ***Segment number*** 37–42. Two chaetae per bundle throughout, absent in XII in mature specimens. ***Chaetae*** straight with slight proximal bend; in anterior segments, slight distal bend in opposite direction of proximal bend, i.e., chaetae faintly sigmoid; in proximal segments, chaetae distally straight. Chaetae in preclitellar bundles 37.5–42 mm long, diameter 5 mm, 27.5–32.5 mm in postclitellar segments, diameter 5 mm. ***Head pore*** mid-dorsally on prostomium. ***Epidermal gland cells*** gray, three to four transverse rows per segment, the cells nearly rectangular and arranged in regular pattern (Fig. 3E). ***Clitellum*** in XII–1/2XIII, inconspicuous thickening, cells ca 5–9 mm high, girdle-shaped (Fig. 3I, J), hyalocytes and granulocytes in reticulate arrangement with hyalocytes taking larger proportion dorsally (Fig. 3I). ***Body wall*** 25–37.5 mm thick.

**Figure 2.**
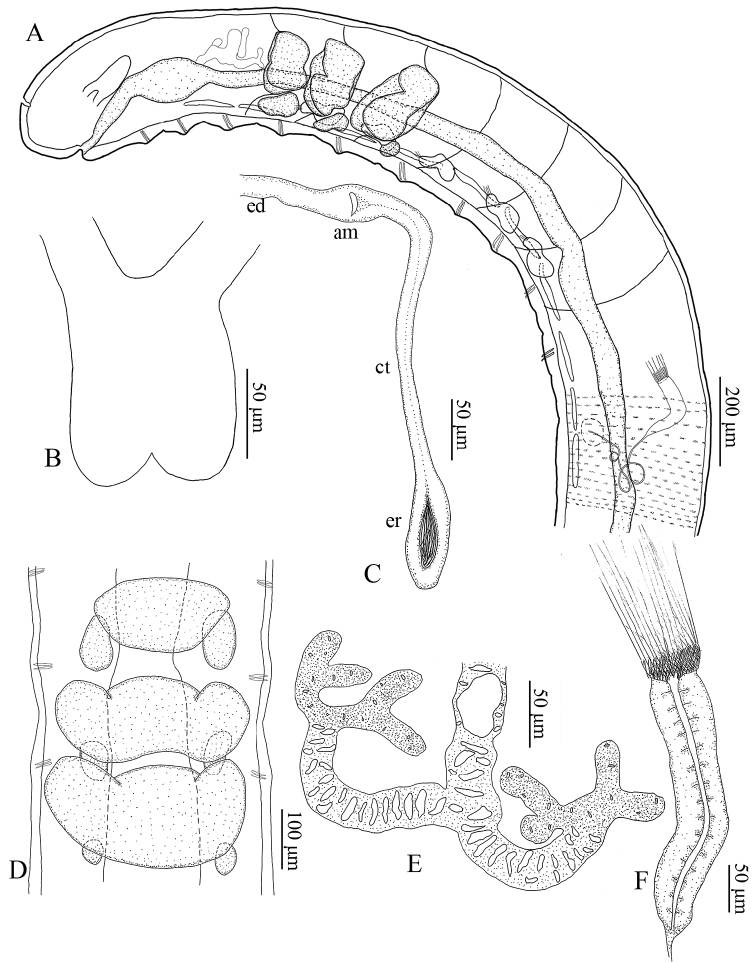
*Hemienchytraeus wuhanensis* sp. nov. **A** anterior body region, anterior 13 segments, lateral view, schematic **B** brain **C** spermatheca; am, ampulla; ct, connecting tube; ed, ectal duct; er, ental reservoir **D** pharyngeal glands **E** oesophageal appendage **F** sperm funnel.

**Figure 3.**
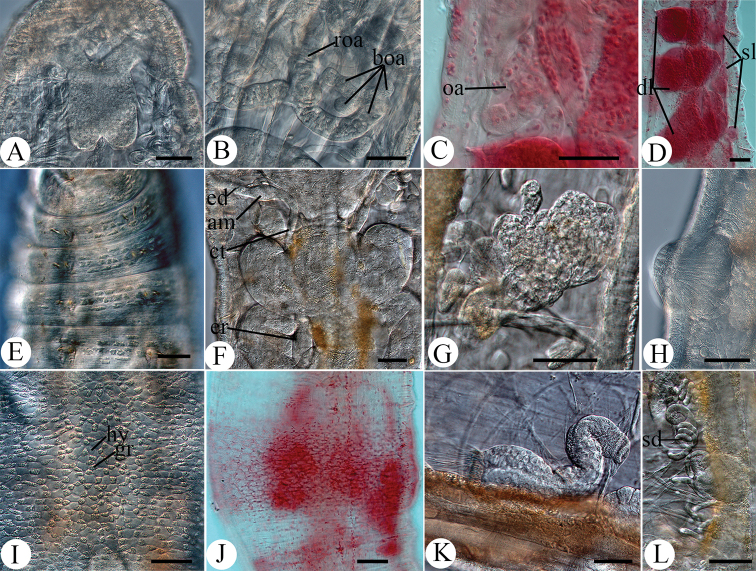
Micrographs of *Hemienchytraeus wuhanensis* sp. nov. **A, B, E–I, K, L***in vivo***C, D, J** fixed **A** brain **B** dorsal view of oesophageal appendage **C** lateral view of oesophageal appendage **D** pharyngeal glands **E e**pidermal gland cells in II–V ventrally **F** spermathecae and pharyngeal glands **G** nephridia in 7/8, anteseptale bottom-left **H** male glandular bulb, slightly everted **I** dorsal view of clitellum **J** ventral view of clitellum **K** sperm funnel **L** sperm duct and musculature of male copulatory organ Abbreviations: roa, root of oesophageal appendage; boa, branches of oesophageal appendage; oa, oesophageal appendage; sl, secondary pharyngeal gland lobes; dl, dorsal lobes of pharyngeal gland; am, ampulla; ct, connecting tube; ed, ectal duct; er, ental reservoir; hy, hyalocyte; gr, granulocyte; sd, sperm duct. Scale bars: 50 μm.

***Brain*** about as long as wide (117 mm long, 93 mm wide, *in vivo*), slightly indented anteriorly, deeply incised posteriorly (Figs 2B, 3A). ***Oesophageal appendage*** arising from mid-dorsal region of pharynx in III as an unpaired root with large proximal chamber; following section longer than proximal chamber, with thick, meandering canal; two primary branches, longer than root, with smaller canal; each primary branch bifurcating into two short, secondary branches; each secondary branch bifurcating into four or more tertiary branches, the latter difficult to distinguish. Secondary and tertiary branches of same diameter, thinner than primary branches (Figs 2E, 3B, C). All three pairs of ***pharyngeal glands*** united dorsally, primary ventral lobes in V and VI. Three pairs of secondary pharyngeal gland lobes in V, VI and VII, small in VII (Figs 2D, 3D). ***Dorsal vessel*** from XII–XIII, blood colorless.

Five pairs of preclitellar ***nephridia*** from 5/6 to 9/10 (Fig. 2A); each about 160 mm long and 60 mm wide (*in vivo*). Anteseptale globular, with minute and numerous brownish granules at periphery; funnel orientated obliquely ventrad, with small and narrow anterior projection; postseptale elongate, ca twice as long as anteseptale. Efferent duct originating from the middle of the postseptale (Fig. 3G).

***Seminal vesicle*** absent, cysts dorsally in XI. ***Sperm funnels*** cylindrical, tapering distad, well developed, ca 150–250 mm long and 40 mm at collar (*in vivo*). Collar distinct, somewhat narrower than funnel body (Figs 2F, 3K). Spermatozoa ca 140 mm long, heads ca 20 mm long (*in vivo*). Sperm ducts elongate, diameter ca 6 mm, loose or tight coils in XII–XIII (Fig. 3L). ***Male copulatory organs*** with distinct musculature, male glandular body globular, ca 85 µm in diameter (*in vivo*). No accessory copulatory glands (Fig. 3H).

***Spermathecae*** free, not attached to oesophagus. Ectal pores laterally at 4/5, without ectal gland. Ectal ducts ca 400–500 mm long and 20–26 mm wide (*in vivo*), with distinct ampullar dilatation in V. Connecting tube between ampulla proper and ental reservoir thinner than ectal duct, extending into VI or VII, ending in a small, elongately ellipsoid ental reservoir of 88–128 µm length and 30–50 µm width (*in vivo*), empty or with spermatozoa (Figs 2C, 3F). One mature egg or 3–4 immature eggs at a time.

##### Molecular results

COI sequences of five paratype specimens of *H. wuhanensis* sp. nov. were successfully acquired and submitted to GenBank with accession numbers. This is the fourth species of *Hemienchytraeus* of which DNA sequences are available (Table 1), the other three being *H. quadratus*, *H. koreanus*, and *H. jeojunensis* Dózsa-Farkas & Hong, 2010, all from South Korea. Clear genetic gaps were observed among the four species with high interspecific distances (7.0–21.9%) and low intraspecific distances (0%) among *H. wuhanensis* sp. nov. specimens based on the K2P distances of COI sequences (Table 2). Interestingly, among the three species from South Korea, the one with lowest genetical distance to *H. wuhanensis* sp. nov., *H. koreanus*, is also the one which is most similar morphologically to the new species (see below).

**Table 1.** List of *Hemienchytraeus* specimens for molecular analyses with collection data and GenBank accession.

Species	Collection information	Specimen ID	Accession number
*H. wuhanensis*-1	Mt Luojia, China	HBO201904011	MW000758
*H. wuhanensis*-2	Mt Luojia, China	HBO201904012	MW000759
*H. wuhanensis*-3	Mt Shizi, China	HBO201904005	MW000760
*H. wuhanensis*-4	Mt Shizi, China	HBO201904006	MW000761
*H. wuhanensis*-5	Mt Shizi, China	HBO201904007	MW000762
*H. quadratus*-1	Mt Hallasan, Korea	1000	MG252159
*H. quadratus*-2	Mt Hallasan, Korea	991	MG252158
*H. koreanus*-1	Mt Hallasan, Korea	1131	MG252157
*H. koreanus*-2	Mt Hallasan, Korea	1005	MG252156
*H. koreanus*-3	Mt Hallasan, Korea	1004	MG252155
*H. koreanus*-4	Mt Hallasan, Korea	1003	MG252154
*H. koreanus*-5	Mt Hallasan, Korea	1002	MG252153
*H. jeonjuensis*	Mt Hallasan, Korea	1115	MG252152

**Table 2.** Genetic distances of four *Hemienchytraeus* species (K2P).

		1	2	3	4	5	6	7	8	9	10	11	12
1	*H. wuhanensis*-1												
2	*H. wuhanensis*-2	0.000											
3	*H. wuhanensis*-3	0.000	0.000										
4	*H. wuhanensis*-4	0.000	0.000	0.000									
5	*H. wuhanensis*-5	0.000	0.000	0.000	0.000								
6	*H. quadratus*-1	0.200	0.200	0.200	0.200	0.200							
7	*H. quadratus*-2	0.198	0.198	0.198	0.198	0.198	0.007						
8	*H. koreanus*-1	0.070	0.070	0.070	0.070	0.070	0.216	0.216					
9	*H. koreanus*-2	0.070	0.070	0.070	0.070	0.070	0.216	0.216	0.000				
10	*H. koreanus*-3	0.070	0.070	0.070	0.070	0.070	0.216	0.216	0.000	0.000			
11	*H. koreanus*-4	0.070	0.070	0.070	0.070	0.070	0.216	0.216	0.003	0.003	0.003		
12	*H. koreanus*-5	0.072	0.072	0.072	0.072	0.072	0.213	0.213	0.002	0.002	0.002	0.005	
13	*H. jeonjuensis*	0.219	0.219	0.219	0.219	0.219	0.189	0.191	0.214	0.214	0.214	0.211	0.211

## Remarks

Three non-sexual characters have been shown to be very useful for the distinction of *Hemienchytraeus* species: oesophageal appendage (branching pattern, relative branch length), secondary pharyngeal gland lobes (number, position, size), and preclitellar nephridia (number, position) (Schmelz et al. 2009). Indeed, these three characters in *H. wuhanensis* suffice to distinguish it from all other species, even from those with an incomplete description, because details of the oesophageal appendage are known in all species, the only exception being *H. brasiliensis* (Cognetti, 1900), a species of uncertain identity (*incertae sedis*) according to Schmelz and Römbke (2005). Further useful characters include the origin of the dorsal blood vessel, presence/absence of a seminal vesicle, shape and size of spermathecae, sperm funnels and male glandular bulbs, and distribution pattern of clitellar gland cells; the latter is fully known only in recently described species.

Considering the three above-mentioned non-sexual diagnostic characters, the new species is most similar to *H. loksai* Dózsa-Farkas, 1989, which also has an oesophageal appendage with tertiary branches, three pairs of secondary pharyngeal gland lobes in V, VI, VII, and five pairs of preclitellar nephridia, from 5/6 to 9/10. However, in *H. loksai* the secondary pharyngeal glands increase in size from IV to VII. The species was described from Ecuador and has been recorded from China (Xie et al. 1999). Further conspicuous differences of *H. loksai* from the new species include larger body size (length >12 mm, 49–55 segments), a postclitellar origin of the dorsal blood vessel, larger spermathecae (extending to IX–X), very large sperm funnels (up to 800–900 µm long), and a huge seminal vesicle (extending into XIV–XVII).

One more species of *Hemienchytraeus* has oesophageal appendages with tertiary branches, i.e., *H. brachythecus*Xie et al., 1999. This species is also similar to the new species in the absence of a seminal vesicle. Conspicuous differences of *H. brachythecus* include a very short spermatheca, confined to V, two pairs of secondary pharyngeal gland lobes in V and VI, and first pair of preclitellar nephridia in 6/7.

Three pairs of secondary pharyngeal gland lobes are also known in *H. koreanus* Dózsa-Farkas & Hong, 2010, and in *H. siljae* Schmelz & Römbke, 2005. *H. koreanus* resembles the new species also in the position of the preclitellar nephridia (5/6–9/10) and in a girdle-shaped clitellum. Conspicuous differences of *H. koreanus* include a postclitellar origin of the dorsal blood vessel, large spermathecae, and the presence of a seminal vesicle.

*H. siljae* resembles the new species in several characters, for example the girdle-shaped clitellum, the absence of a seminal vesicle, and the approximate shape and size of spermathecae and sperm funnels. Conspicuous differences include a more posterior origin of the dorsal blood vessel (XIV), four pairs of preclitellar nephridia, from 6/7 to 9/10, and an oesophageal appendage with three elongate secondary branches on each side, without tertiary branches.

A comparison of these four species with the new one is presented in Table 3.

**Table 3.** Comparison of *H. wuhanensis* sp. nov. with similar species.

	***H. wuhanensis* sp. nov.**	***H. brachythecus* Xie et al., 1999**	***H. siljae* Schmelz et al., 2005**	***H. loksai* Dózsa-Farkas, 1989**	***H. koreanus* Dózsa-Farkas & Hong, 2010**
Secondary pharyngeal gland lobes	3 pairs, V–VII	2 pairs, V–VI	3 pairs, V–VII	3 pairs, V–VII	3 pairs, V–VII
Oesophageal appendage	4 or more tertiary branches	3–4 tertiary branches	4–5 elongate secondary branches	3–4 tertiary branches	5–6 secondary branches
Preclitellar nephridia	5; 5/6–9/10	5; 6/7–10/11	4; 6/7–9/10	5; 5/6–9/10	5; 5/6–9/10
Sperm funnel: shape; length:width ratio	Cylindrical; 4–6:1	Subspherical; 1.6–2:1	Cone-shaped; 4–6:1	Cone-shaped; 9:1	Cone-shaped; 5–6:1
Spermathecae, extension	VI–VII	V	VI–VIII	IX–X	VIII–X
Seminal vesicle	Absent	Absent	Absent	XII–XIV	XII–XIII
Epidermal gland cells	3–4 rows per segment	Scarce	4–5 rows in preclitellar segments	6–8 rows per segment	3–4 rows per segments

With the description of *H. wuhanensis* sp. nov., there are now seven species of *Hemienchytraeus* known from China. Two of them were originally described from China and have not been recorded elsewhere: *Hemienchytraeus planisetosus*Xie et al., 1999 and *Hemienchytraeus brachythecus*Xie et al., 1999. The other four species were originally described from different countries, and the records from China require confirmation, for various reasons.

The record of *Hemienchytraeus stephensoni* Cognetti, 1927, from Hunan Province (Xie et al. 1999) was rejected by Schmelz and Collado (2007), after a type-based revision of this nominal species (Schmelz and Collado 2007), which narrowed the range of variation of taxonomically important characters. *Hemienchytraeus stephensoni* sensu Xie et al. (1999) may in fact be a species new to science. *Hemienchytraeus stephensoni* was originally described from India as *Enchytraeus cavicola* Stephenson, 1924; see Schmelz and Collado (2007) for the nomenclatural history.

*Hemienchytraeus bifurcatus* Nielsen & Christensen, 1959 originally described from Denmark, has been considered a “*species inquirenda*” (Schmelz and Römbke 2005), because the original description is incomplete with respect to secondary pharyngeal gland lobes, preclitellar nephridia, and details of the clitellum. A validation of *H. bifurcatus* is difficult because type material is lost, and efforts to obtain fresh material at the type locality have so far been unsuccessful (Schmelz and Römbke 2005). Hence, the records of this species from China (Liang and Xie 1992; Wang and Liang 2002) cannot be confirmed; those specimens may just as well belong to a new species.

The redescription of *H. loksai* by Xie et al. (1999) based on material from Hunan Province, China, agrees with the original description in conspicuous details (e.g., size of seminal vesicle and sperm funnels) but lacks information on the secondary pharyngeal gland lobes; furthermore, the first preclitellar nephridia are in 6/7, not in 5/6 as originally described. Material of *H. loksai* sensu Xie et al. (1999) should be reinvestigated to confirm the species identity of the specimens.

Finally, *H. theae* Prabhoo, 1961 described from India, and recorded from China by Liang and Xie (1992), was originally insufficiently described: secondary pharyngeal gland lobes, number and position of nephridia, details of the clitellum, and origin of the dorsal blood vessel are unknown. Reinvestigation of the type material present at the Zoological Survey of India (Prabhoo 1961) and comparison with the material underlying the record of Liang and Xie (1992) would be necessary to confirm the species identity of the Chinese specimens.

Despite these taxonomic uncertainties, the presence of at least seven species of *Hemienchytraeus* in China is beyond doubt. Actually, many more species of *Hemienchytraeus* are to be expected in this country, in view of the preference for tropical or subtropical soils of this globally distributed genus.

## Supplementary Material

XML Treatment for Hemienchytraeus wuhanensis
